# Clonal analysis reveals remarkable functional heterogeneity during hematopoietic stem cell emergence

**DOI:** 10.1038/cr.2017.64

**Published:** 2017-04-28

**Authors:** Hui Ye, Xiaobo Wang, Zongcheng Li, Fan Zhou, Xianlong Li, Yanli Ni, Weijing Zhang, Fuchou Tang, Bing Liu, Yu Lan

**Affiliations:** 1 State Key Laboratory of Proteomics, Translational Medicine Center of Stem Cells, 307-Ivy Translational Medicine Center, Laboratory of Oncology, Affiliated Hospital, Academy of Military Medical Sciences, Beijing 100071, China; 2 Institute of Hematology, School of Medicine, Jinan University, Guangzhou, Guangdong 510632, China; 3 The Hospital of NO 61016 Troops of PLA, Beijing 102202, China; 4 Biodynamic Optical Imaging Center, College of Life Sciences, Peking University, Beijing 100871, China; 5 Affiliated Hospital, Academy of Military Medical Sciences, Beijing 100071, China; 6 Peking-Tsinghua Center for Life Sciences, Peking University, Beijing 100871, China; 7 Ministry of Education Key Laboratory of Cell Proliferation and Differentiation, Beijing 100871, China; 8 Center for Molecular and Translational Medicine (CMTM), Beijing 100101, China; 9 State Key Laboratory of Experimental Hematology, Institute of Hematology and Blood Diseases Hospital, Chinese Academy of Medical Sciences, Tianjin 300020, China

## Dear Editor,

In conventional opinion, hematopoietic stem cells (HSCs) are alike, possessing robust self-renewal and multilineage differentiation capacity. However, growing evidence has revealed striking functional heterogeneity among individual HSCs, particularly in the aspect of lymphomyeloid output^1,2,3^. Four subtypes of HSCs with distinct differentiation patterns have been identified and designated as α-, β-, γ-, and δ-HSCs^4^. Only lymphoid-deficient (α) and lymphomyeloid-balanced (β) HSCs bear durable engraftment potential and extensive self-renewal activity^5^. Although differentiation program of individual HSCs can be stably self-propagated, occasional occurrence of inter-conversions among programs has also been observed^5^. HSC heterogeneity exhibits developmental stage-related fluctuation, especially regarding the prevalence of α-HSCs, being minor in fetal liver but high in aging bone marrow (BM)^5^. The arising concept of HSC heterogeneity leads to a series of key questions, as to its embryonic origin, regulatory mechanisms, and relationship with leukemogenesis and therapeutics.

The first transplantable adult-like HSCs become detectable in the aorta-gonad-mesonephros (AGM) region of mice around embryonic day (E) 10.5^6^. At this time, the AGM region contains at least two types of pre-HSCs^7^, characterized by a high expression level of CD201^8^. Pre-HSCs possess abundant expression of endothelial signature genes and can develop into *bona fide* HSCs after co-culture with stromal cells and cytokines *in vitro*. It is of great interest but remains unknown whether and to what extent HSC heterogeneity presents when pre-HSCs and HSCs emerge in mid-gestation embryos.

Approximately 3 AGM regions at E11.0 (40-44 somite pairs, sp) contain only one mature HSC. This frequency markedly increases to one HSC per AGM at E11.5 (45-48 sp)^9^. To ensure rigorous clonal analysis, we dissected E11 AGM tissues, with each AGM equally divided into 2 (E11.0) or 3 (E11.5) aliquots for subsequent direct transplantation into lethally irradiated adult mice. Recipients demonstrating ≥ 1% donor-derived white blood cells (WBCs) in peripheral blood after a minimum of 4 months were considered to be long-term reconstituted (Supplementary information, Figure S1A). Out of 139 recipients, 54 demonstrated successful long-term (> 4 months) reconstitution. 14 recipients repopulated by both aliquots from 7 E11.0 embryos were ruled out, and the remaining 40 were used for further analyses (Supplementary information, Figure S1B). The results are highly reminiscent of previous data using limiting dilution assay^9,10^, ensuring the reconstitution at a clonal level.

**Figure 1:**
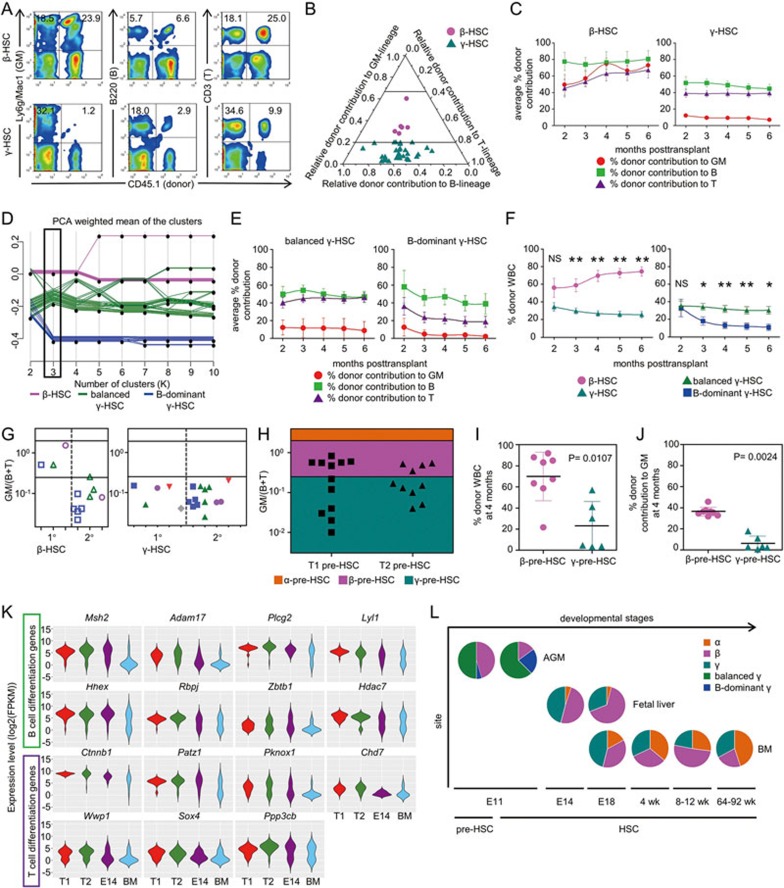
Characterization of HSC and pre-HSC subtypes in E11 AGM. **(A)** Representative FACS plots of β-HSCs and γ-HSCs 4 months post-transplantation. **(B)** Ternary plot showing relative donor contributions to different lineages in 40 reconstituted recipients 4 months post-transplantation. The criteria to discriminate α-, β-, and γ-HSCs are indicated by the two horizontal lines. β-HSC-derived cells display balanced GM/(B+T) contribution ratios (0.25–2.0) shown in the middle (magenta) and γ-HSC-derived cells display low GM/(B+T) contribution ratios (< 0.25) shown in the bottom (blue-green). **(C)** Donor contributions of β-HSCs and γ-HSCs to GM (red), B cell (green), and T cell (purple) lineages 2-6 months post-transplantation. Data shown are means ± SEM. **(D)** K-means analysis of relative lineage contributions of HSCs shown as principal component analysis (PCA) values (from the 4-month data). When the normalized relative lineage contributions are assigned to 2 clusters (K = 2), 40 clonal HSCs are segregated into mainly β and γ subtypes. γ-HSCs are further segregated into 2 distinct populations when K = 3 (boxed), indicative of their distinct differentiation patterns. **(E)** Donor contributions of two subtypes of γ-HSCs to GM (red), B cell (green), and T cell (purple) lineages 2-6 months post-transplantation. Data shown are means ± SEM. **(F)** Donor contributions of different subtypes of HSCs to WBCs in the peripheral blood of recipients 2-6 months post-transplantation. Data shown are means ± SEM. NS, not significant; *P < 0.05; **P < 0.01. **(G)** GM/(B+T) values in the primary (3 β-HSCs and 5 γ-HSCs) and related secondary recipients 4 months post-transplantation. The criteria to discriminate α-, β-, and γ-HSCs are indicated by the two horizontal lines. Identical symbols represent paired primary and secondary repopulated mice. **(H)** GM/(B+T) ratios of T1 and T2 pre-HSCs in E11 AGM (*n* = 22). The two horizontal lines distinguish three pre-HSC subtypes: α-pre-HSCs (orange), β-pre-HSCs (magenta) and γ-pre-HSCs (blue-green). All data are collected over 4 months post-transplantation. **(I**, **J)** Donor contributions of β-pre-HSCs and γ-pre-HSCs to WBCs **(I)** or GM lineage **(J)** in the peripheral blood of recipients 4 months post-transplantation. Each point represents an individual mouse. Horizontal bars indicate the mean values. **(K)** Violin plots of 15 representative lymphoid differentiation-related genes with significantly higher expression levels in pre-HSCs (T1 and T2) and fetal liver HSCs (E14), than in adult HSCs (BM). **(L)** Pie charts showing subtypes of pre-HSCs and HSCs across developmental stages. Data of fetal liver and BM are from the study by the Eaves group^5^. wk, weeks.

Designation of HSC subtypes is mainly based on calculating the ratio of donor-derived contribution to granulocytes (G) plus monocytes (M) versus to B and T lymphocytes (GM/(B+T)). Namely, α- and β-HSCs are defined as GM/(B+T) ≥ 2 and 0.25-2, respectively. The left populations (GM/(B+T) ≤ 0.25) are further divided according to whether donor contribution to GM is ≥ 1% (γ-HSCs) or not (δ-HSCs)^4,5^. Our analysis showed that E11 AGM regions contained only two HSC subtypes. The majority (34/40, 85%) were γ-HSCs and the remaining 15% (6/40) were β-HSCs (Figure 1A-1B). The kinetics of average lineage contributions showed obviously distinct patterns between β-HSCs and γ-HSCs from 2 to 6 months post-transplantation, particularly regarding contributions to the GM lineage (Figure 1C).

Interestingly, unlike HSCs in adult BM^4^, some B lymphoid lineage-dominant γ-HSCs were observed in E11 AGM (Figure 1B). Using the k-means algorithm, 34 γ-HSCs were further separated into 2 distinct clusters (Figure 1D). Based on the corresponding distribution on the ternary graph, 9 were designated as B lymphoid lineage-dominant (B-dominant) γ-HSCs, with GM/(B+T) ≤ 0.25 and B/(GM+T) ≥ 1.5, and the other 25 were defined as lymphoid lineages-balanced (balanced) γ-HSCs (Supplementary information, Figure S2A). Compared to balanced γ-HSCs, B-dominant γ-HSCs showed impaired T lymphoid and more severely deficient myeloid differentiation potentials (Figure 1E).

Similar to what was observed in adult BM^4^, embryonic β-HSCs demonstrated a significantly higher reconstitution ability than γ-HSCs. Moreover, B-dominant γ-HSCs displayed a remarkably lower reconstitution ability than balanced γ-HSCs (Figure 1F and (Supplementary information, Figure S2B). Unlike balanced γ-HSCs showing a sustained reconstitution, B-dominant γ-HSCs manifested decreased reconstitution levels over time (Figure 1F). These data suggest that the most limited repopulating potential of HSCs during midgestation is related to their compromised ability to differentiate into myeloid and T lymphoid lineages.

In adult BM, β-HSCs display extensive self-renewal activity, whereas γ-HSCs fail to repopulate secondary recipients^4,5^. We performed secondary transplantations using 3 primary recipients of β-HSCs and 5 primary recipients of balanced γ-HSCs. Cells from the 3 primary recipients of β-HSCs reconstituted 9 of 18 secondary recipients (Supplementary information, Figure S2C-S2D). Unexpectedly, only 1 secondary recipient displayed a β-HSC differentiation pattern, whereas the other 8 showed a balanced γ-HSC differentiation pattern (Figure 1G and Supplementary information, Figure S2D). Interestingly, cells from 4 out of 5 primary recipients of balanced γ-HSCs reconstituted 13 of 23 secondary recipients (Supplementary information, Figure S2C-S2D). Among them, 11 maintained the balanced γ-HSC pattern, and the other 2 showed a B-dominant γ-HSC feature, highly suggesting preservation of differentiation programs of balanced γ-HSCs (Figure 1G and Supplementary information, Figure S2D). Thus, embryonic β-HSCs can give rise to both β- and γ-HSCs, and the embryonic γ-HSCs can only give rise to γ-HSCs, supporting a higher hierarchy of β-HSCs than that of γ-HSCs, in line with what was observed in BM^4,5^. In contrast to adult BM^4,5^, E11 AGM regions contain predominantly γ-HSCs but less β-HSCs. Moreover, embryonic balanced γ-HSCs showed a much stronger self-renewal potential than those in adults, whereas embryonic β-HSCs showed a limited preservation of their differentiation pattern upon secondary transplantation.

We have recently established a strategy to efficiently isolate individual AGM-derived pre-HSCs^8^. We performed co-culture/transplantation assays initiated with single pre-HSCs from E11 AGM. The 22 repopulated recipients, including 12 receiving type 1 (T1, CD45-negative) and 10 receiving type 2 (T2, CD45-positive) pre-HSCs, displayed either a β or γ differentiation pattern (Figure 1H and Supplementary information, Figure S2E). There were totally 10 β-pre-HSCs (6 T1 and 4 T2), 10 balanced γ-pre-HSCs (5 T1 and 5 T2), and only 2 B-dominant γ-pre-HSCs (Figure 1H and Supplementary information, Figure S2F). β-pre-HSCs showed significantly higher reconstitution ability than γ-pre-HSCs (Figure 1I). The donor contributions to GM lineage in recipients of β-pre-HSCs were dramatically higher than those in recipients of γ-pre-HSCs at 4 months post-transplantation, and this was not seen regarding donor contributions to B and T lymphoid lineages (Figure 1J and Supplementary information, Figure S2G). Notably, α- or δ-pre-HSCs were undetectable, consistent with results of direct transplantation with AGM HSCs (Supplementary information, Figure S2F).

Finally, we analyzed a recently reported dataset of singe-cell RNA-seq of multiple HSC-competent populations^8^. Out of 77 hematopoietic lineage-differentiation genes, 36 genes showed significantly differential expression between embryonic (pre-HSCs and fetal liver HSCs) and adult populations, most of which (29/36) displayed higher expression levels in the former (Supplementary information, Figure S2H). Among these 29 genes, 17 are related to lymphoid lineage differentiation and the expression level distributions of these genes in the fetal liver HSCs were usually either like those in pre-HSCs or similar to those in BM HSCs, suggesting a gradual change of the differentiation programs along with development (Figure 1K).

In summary, our study unveils a heterogeneity in pre-HSC/HSC composition in E11 mouse embryos, indicating that functional heterogeneity in HSCs exists from the very beginning of embryonic HSC emergence and persists throughout the whole lifespan^4,5^ (Figure 1L). This study reveals at least two major HSC subtypes closely associated with the wall of large arteries during the course of HSC emergence, prior to the colonization of mature HSCs to fetal liver. The mechanism for the absence of α-HSCs deserves further investigations, which might be related to the influence of the microenvironment^5^. In addition, we are searching for candidate surface markers that can be used to isolate certain subtypes of pre-HSCs and HSCs in various hemogenic and hematopoietic niches.

## Supplementary Information

Supplementary information, Figure S1 Experimental design for clonal HSC and single pre-HSC analysis.

Supplementary information, Figure S2 Functional heterogeneity of HSCs and pre-HSCs in E11 AGM.
