# Supplementary material for: Clonal analysis reveals remarkable functional heterogeneity during hematopoietic stem cell emergence
Source: Cell Res. 2017 Apr 28;27(8):1065–8. doi: 10.1038/cr.2017.64 (PMC5539347; doi:10.1038/cr.2017.64)
Supplement: Supplementary information, Figure S1 — Experimental design for clonal HSC and single pre-HSC analysis. [file cr201764x1.pdf]

Clonal analysis reveals remarkable functional heterogeneity during hematopoietic stem cell emergence

Figure S1

A

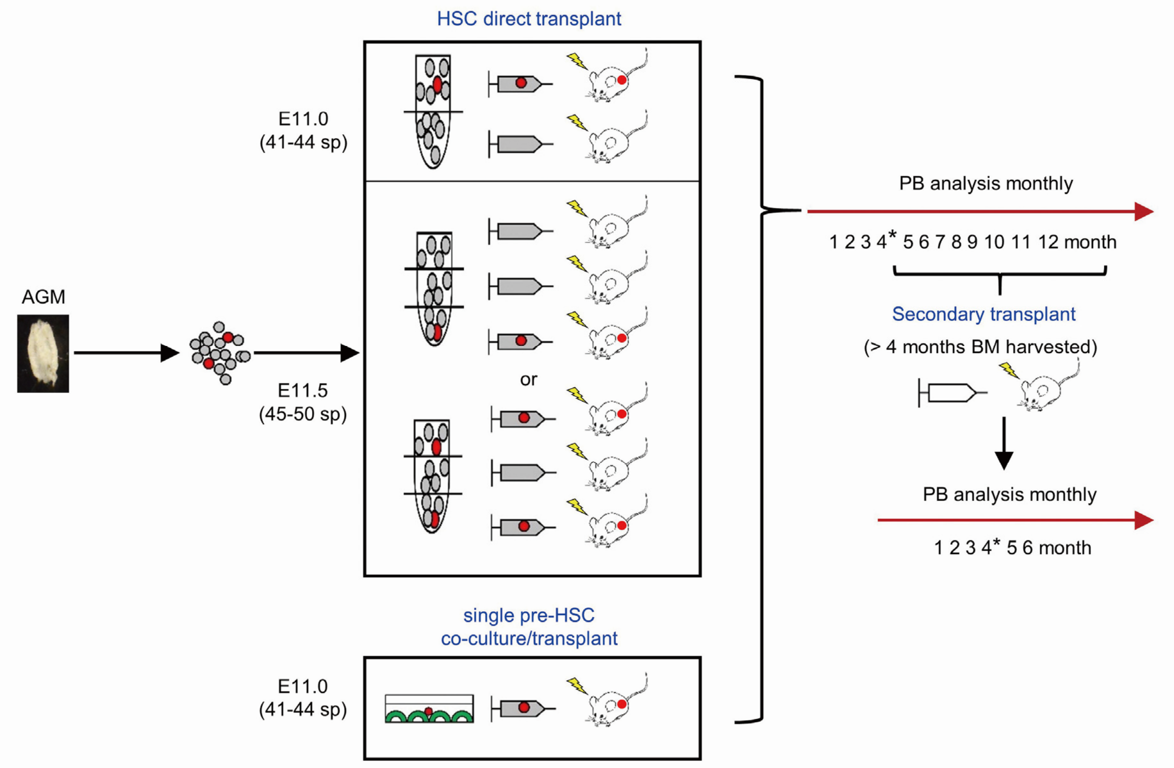

B

Developmental stage	Independent experiments	Reconstituted /total aliquots for each embryo	Number of donor embryos	Number of recipients	Reconstituted recipients	Reconstituted /total recipients
E11.0 (41-45 sp)	6	0/2	22	44	0	35/100 (35.0%)
		1/2	21	42	21	
		2/2	7	14	14 (not included)	
E11.5 (46-50 sp)	3	0/3	1	3	0	19/39 (48.7%)
		1/3	5	15	5	
		2/3	7	21	14	
		3/3	0	0	0 (not included)	
Total	9	/	63	139	40	54/139 (38.8%)

Supplementary information, Figure S1. Experimental design for clonal HSC and single pre-HSC analysis. (A) The transplantable HSCs were obtained from 41-50 sp AGM region, and single pre-HSCs were obtained from FACS sorting of 41-45 sp AGM region. After primary transplantation, the peripheral blood (PB) of recipients was collected and analyzed monthly (up to 12 months). Recipients from secondary transplantation were followed up to 6 months. 4*, 4 months post-transplantation as the time point for reconstitution judgment. **(B)** Details of the reconstituted mice by direct transplantation.
