# Supplementary material for: Clonal analysis reveals remarkable functional heterogeneity during hematopoietic stem cell emergence
Source: Cell Res. 2017 Apr 28;27(8):1065–8. doi: 10.1038/cr.2017.64 (PMC5539347; doi:10.1038/cr.2017.64)
Supplement: Supplementary information, Figure S2 — Functional heterogeneity of HSCs and pre-HSCs in E11 AGM. [file cr201764x2.pdf]

# Figure S2

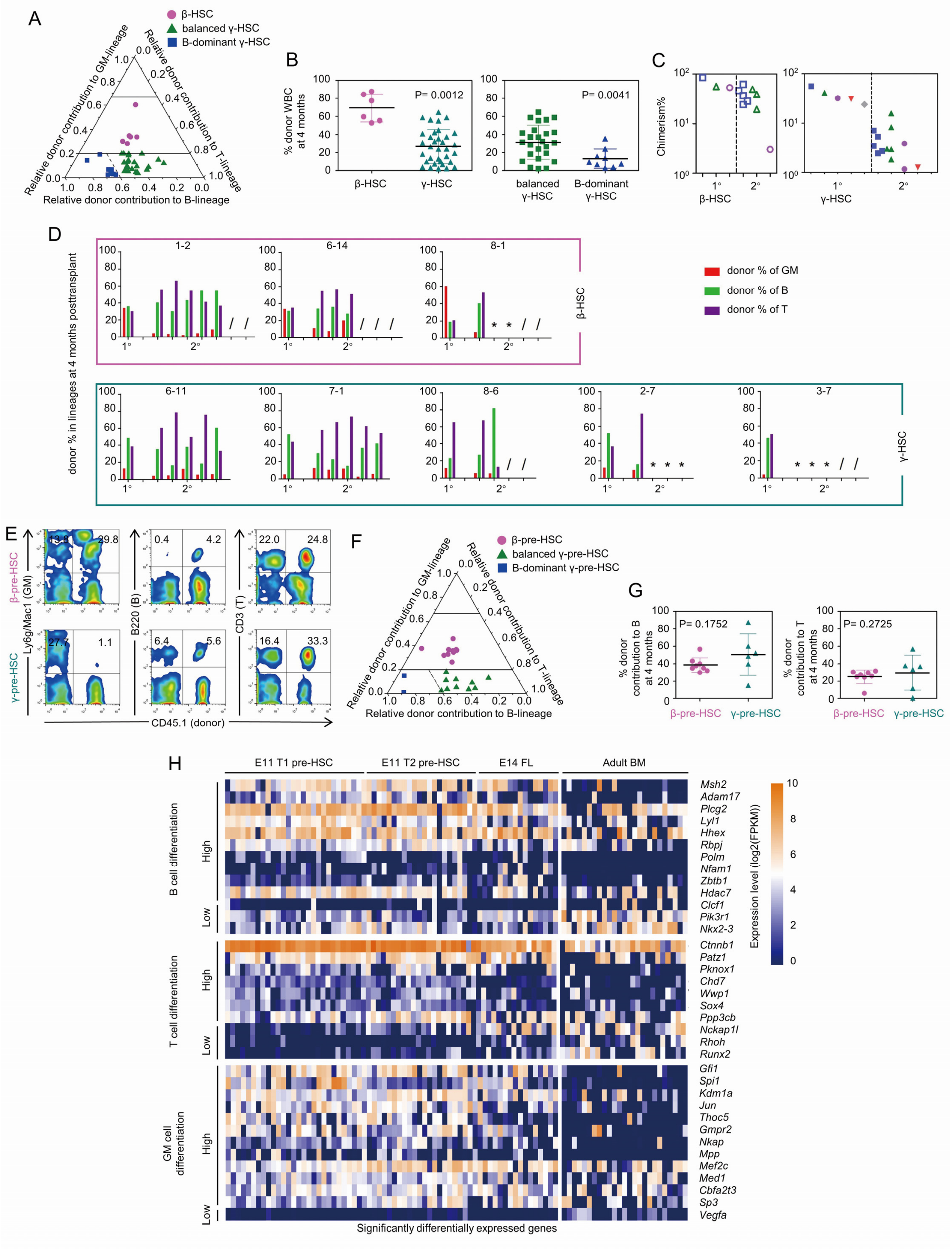
A
β-HSC
balanced γ-HSC
B-dominant γ-HSC
Relative donor contribution to GM-lineage
Relative donor contribution to T-lineage
Relative donor contribution to B-lineage
B
% donor WBC at 4 months
P= 0.0012
P= 0.0041
β-HSC
γ-HSC
balanced γ-HSC
B-dominant γ-HSC
C
Chimerism%
1°
2°
β-HSC
γ-HSC
D
donor % in lineages at 4 months posttransplant
1-2
6-14
8-1
β-HSC
donor % of GM
donor % of B
donor % of T
6-11
7-1
8-6
2-7
3-7
γ-HSC
E
β-pre-HSC
γ-pre-HSC
Ly6g/Mac1 (GM)
B220 (B)
CD3 (T)
CD45.1 (donor)
F
β-pre-HSC
balanced γ-pre-HSC
B-dominant γ-pre-HSC
Relative donor contribution to GM-lineage
Relative donor contribution to T-lineage
Relative donor contribution to B-lineage
G
% donor contribution to B at 4 months
P= 0.1752
% donor contribution to T at 4 months
P= 0.2725
β-pre-HSC
γ-pre-HSC
H
E11 T1 pre-HSC
E11 T2 pre-HSC
E14 FL
Adult BM
B cell differentiation
T cell differentiation
GM cell differentiation
High
Low
Msh2
Adam17
Plcg2
Lyl1
Hhex
Rbpj
Polm
Nfam1
Zbtb1
Hdac7
Clcf1
Pik3r1
Nkx2-3
Ctnnb1
Patz1
Pknox1
Chd7
Wwp1
Sox4
Ppp3cb
Nckap1l
Rhoh
Runx2
Gfi1
Spi1
Kdm1a
Jun
Thoc5
Gmpr2
Nkap
Mpp
Mef2c
Med1
Cbfa2t3
Sp3
Vegfa
Expression level (log2(FPKM))
Significantly differentially expressed genes

**Supplementary information, Figure S2. Functional heterogeneity of HSCs and pre-HSCs in E11 AGM. (A)** Ternary plot showing the distribution of individual relative lineage contribution of 40 reconstituted recipients at 4 months post-transplantation. The criteria to discriminate α-, β-, and γ-HSCs is indicated by the two horizontal lines. Dashed line further distinguishes balanced γ-HSCs and B-dominant γ-HSCs according to k-means analysis. Three subtypes of HSCs are shown in different colors and shapes. **(B)** Donor contributions of different subtypes of HSCs to WBCs in the peripheral blood of recipients at 4 months post-transplantation. Each point represents an individual mouse. Horizontal bars indicate the mean values. p values are indicated. **(C)** WBC chimerism in the primary (3 of β-HSCs and 5 of γ-HSCs) and related secondary recipients at 4 months post-transplantation. Identical symbols represent paired primary and secondary repopulated mice. **(D)** Donor contributions to the GM (red), B cell (green), and T cell (purple) lineages at 4 months post-transplantation in primary (β-HSCs, upper; γ-HSCs, lower) and related secondary recipients. / indicates the recipient died before 4 months post-transplantation. * indicates the recipient with < 1% donor WBCs at 4 months post-transplantation, considered as unsuccessfully reconstituted. **(E)** Representative FACS plots of lineage constitutions in the peripheral blood of recipients of β- and γ-pre-HSCs derived co-cultures at 4 months post-transplantation. **(F)** Ternary plot showing the distribution of individual relative lineage contribution of 22 reconstituted recipients of pre-HSCs collected over 4 months post-transplantation. The criteria to discriminate α-, β-, and γ-pre-HSCs is indicated by the two horizontal lines. Dashed line further distinguishes balanced γ-pre-HSCs and B-dominant γ-pre-HSCs. Three subtypes of pre-HSCs are shown in different colors and shapes. **(G)** Donor contributions of β-pre-HSCs and γ-pre-HSCs to B (left) and T (right) cell lineages at 4 months post-transplantation. Each point represents an individual mouse. Horizontal bars indicate the mean values. p values are indicated. **(H)** Heatmap showing the expression levels of the 36 significantly differentially expressed lineage-differentiation genes between embryonic and adult HSC-competent populations. High and Low to the left of the heatmap indicate the genes that are higher or lower expressed in embryonic cells than in adult cells, respectively.
