# Supplementary material for: Clonal analysis reveals remarkable functional heterogeneity during hematopoietic stem cell emergence
Source: Cell Res. 2017 Apr 28;27(8):1065–8. doi: 10.1038/cr.2017.64 (PMC5539347; doi:10.1038/cr.2017.64)
Supplement: Supplementary information, Data S1 — Materials and Methods [file cr201764x3.pdf]

Supplementary information, Data S1 Materials and Methods

Animals

Mice experiments were approved in the ethics committee of the Affiliated Hospital of Academy of Military Medical Sciences. All experiments used C57BL/6J (B6) background mice. The embryos from the positive detection of vaginal plug were defined as E0 in the day morning. E11 embryos (41-50 sp) obtained from female CD45.2/2 mice crossed with male CD45.1/1 mice or male GFP mice [1] were dissected under the microscope (Leica). Dissection of mouse AGM region and preparation of the single-cell suspension have been described elsewhere [2].

Flow cytometry

Cells were analyzed and sorted by flow cytometer Calibur or Arial 2 (BD). The cells were stained with following antibodies: CD31 (MEC13.3), CD41 (MWReg30), CD45 (30-F11), c-Kit (2B8), CD201 (eBio1560), CD45.1 (A20), CD45.2 (104), Ly-6G (1A8), Mac-1 (M1/70), B220 (RA3-6B2), CD3e (145-2C11), Ter119 (TER-119), Gr-1 (RB6-8C5), CD127 (A7R34), CD4 (GK1.5), CD8a (53-6.7), CD48 (HM48-1), Sca-1 (D7), CD150 (TC15-12F12.2), and Streptavidin APC-eFluor 780. 7-amino-actinomycin D (7-AAD) was used to exclude dead cells. All monoclonal antibodies and 7-AAD were purchased from eBioscience, except for CD31 and CD41 from BD Pharmingen, and CD150 and Ly-6G from BioLegend.

Pre-HSC co-culture assay

The OP9-DL1 cells were cultured properly in a 24-well plate 1-2 days before the FACS-isolated pre-HSCs were seeded individually. After 6 days of co-culture, the descendants of single pre-HSC in each well were harvested respectively for further transplantation. Details of the media and cytokines have been reported previously [3].

Transplantation assay

The freshly prepared or co-cultured cells from mouse AGM regions were injected together with 2×10^4 nucleated fresh bone marrow cells into 8-12 weeks CD45.2/2 female recipients exposed to a split dose of 9 Gy γ-irradiation (^{60}Co). The recipients demonstrating ≥ 1% donor derived WBCs outputs in peripheral blood at 4 months post-transplantation were defined as

reconstituted successfully. Secondary transplant recipients were injected with 1×10^7 bone marrow cells obtained from primary reconstituted mice to investigate HSC self-renewal potential. Peripheral blood cells were collected monthly to analyze precisely the starting pre-HSC/HSC subtype.

Experiments data analysis

Peripheral blood FACS data were analyzed by FlowJo software (Tree Star), and other data were displayed by GraphPad Prism 5 software or Origin 9 software.

Single-cell RNA-seq data analysis

The lineage differentiation genes were collected from gene ontogeny terms in which genes represented definitive lineage regulators. The R package limma was employed for differential expression analysis [4].

Statistical analysis

All experiments data were used SAS 9.1 software to evaluate p value by cochran's T test or Wilcoxon test. Heatmap and violin plots of genes were performed using R language. $p < 0.05$ was considered as statistically significant (the Benjamini and Hochberg method was used for multiple testing adjustment).

References

1 Tan XW, Liao H, Sun L, Okabe M, Xiao ZC, Dawe GS. Fetal microchimerism in the maternal mouse brain: a novel population of fetal progenitor or stem cells able to cross the blood-brain barrier? *Stem cells* 2005; **23**:1443-1452.

2 Li Z, Lan Y, He W *et al.* Mouse embryonic head as a site for hematopoietic stem cell development. *Cell stem cell* 2012; **11**:663-675.

3 Zhou F, Li X, Wang W *et al.* Tracing haematopoietic stem cell formation at single-cell resolution. *Nature* 2016; **533**:487-492.

4 Ritchie ME, Phipson B, Wu D *et al.* limma powers differential expression analyses for RNA-sequencing and microarray studies. *Nucleic Acids Res* 2015; **43**:e47.
